# Alternative Lead ECG Placements

**DOI:** 10.1111/anec.70176

**Published:** 2026-03-28

**Authors:** José Luis Morales‐Arteaga, Amin Meghdadi, Derek Wu, Abdullah Bhuiyan, Nicole Langleben, Cengiz Burak, Emily Wilson, Ashley Pellerito, David Aristizábal, Keanu Razzaghi, Juan Maria Farina, Shyla Gupta, Adrian Baranchuk

**Affiliations:** ^1^ School of Medicine Universidad Panamericana Mexico City Mexico; ^2^ Faculty of Health Sciences Queen's University Kingston Ontario Canada; ^3^ Department of Biomedical Engineering and Health Sciences McMaster University Hamilton Ontario Canada; ^4^ Department of Medicine Queen's University Kingston Ontario Canada; ^5^ Faculty of Life Sciences Queen's University Kingston Ontario Canada; ^6^ Kingston Health Science Centre Ontario Canada; ^7^ Department of Cardiology University of Antioquia Medellín Colombia; ^8^ Faculty of Life Sciences McMaster University Hamilton Ontario Canada; ^9^ Department of Cardiovascular and Thoracic Surgery Mayo Clinic Phoenix Arizona USA; ^10^ Faculty of Medicine University of Ottawa Ottawa Ontario Canada

**Keywords:** arrhythmia, ARVD, Brugada, diagnosis, electrocardiography, ischemia, lead

## Abstract

Electrocardiography (ECG) is a critical diagnostic tool for identifying cardiac conditions. While standard lead positions are widely used to ensure diagnostic accuracy, alternative lead placements have been developed to address specific clinical scenarios. These alternative configurations can overcome physical or technical challenges, enhance rhythm assessment, improve signal quality, and provide greater specificity for certain conditions, ultimately enabling more personalized diagnostic strategies. This paper examines the clinical significance of alternative ECG lead positions, highlighting their advantages, limitations, and potential applications in various clinical settings.

## Graphical abstract

Alternative ECG lead placements can improve diagnostic yield when the standard 12‐lead ECG is suboptimal or infeasible. This review summarizes practical, scenario‐driven modifications (right‐sided, posterior, seated/prone, Lewis, Fontaine, torso‐based, and monitoring configurations) to better detect ischemia, right ventricular disease/ARVD, and inherited arrhythmia syndromes while enabling faster, more adaptable acquisition.

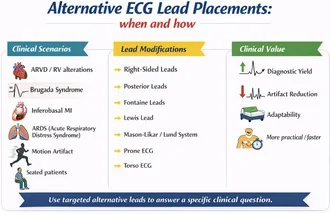

## 1 Introduction

Electrocardiography (ECG) is a cornerstone diagnostic tool in cardiovascular medicine, providing vital insights into the heart's electrical activity. The standard 12‐lead ECG offers a comprehensive view of the heart from multiple angles, but its diagnostic accuracy depends heavily on proper lead placement (Baranchuk et al. 2009). Suboptimal positioning can result in ambiguous findings, potentially leading to misdiagnosis and inappropriate treatment (Baranchuk et al. 2009).

In specific clinical scenarios, alternative lead placements are critical for detecting conditions often missed by standard configurations. For example, adding posterior leads (V7–V9) significantly improves the detection of inferolateral myocardial infarction, enhancing diagnostic sensitivity (Rao et al. 2025). Right ventricular involvement in the setting of inferior STEMI is usually assessed with right‐sided precordial leads (V3R–V4R), and contemporary STEMI guidelines recommend recording these leads whenever right ventricular infarction is suspected (Rao et al. 2025). Non‐standard ECG lead configurations can provide critical diagnostic utility in the detection of cardiac channelopathies, electrolyte disturbances, pharmacologic or toxicologic effects on cardiac conduction, selected clinical conditions, and in patients for whom acquisition of a conventional 12‐lead ECG is unfeasible (Baranchuk et al. 2009). This narrative review examines alternative lead placements, highlighting their diagnostic utilities and clinical significance, offering a practical guide for optimizing ECG interpretations.

## 2 Methods

This narrative review was conducted by identifying and synthesizing published clinical studies and technical reports that evaluated alternative 12‐lead ECG lead positions or torso‐ECG systems in humans. We focused on configurations designed to improve the detection of key diagnostic patterns (e.g., Brugada‐type ST‐segment elevation, epsilon waves in arrhythmogenic right ventricular dysplasia/cardiomyopathy [ARVD/C], atrial activity in supraventricular tachycardia, and ischemic ST‐segment deviations) compared with the conventional 12‐lead ECG.

We performed a structured search of PubMed/MEDLINE from database inception through September 2025 using combinations of the following terms: “Brugada syndrome,” “Brugada‐type ECG,” “arrhythmogenic right ventricular dysplasia,” “arrhythmogenic cardiomyopathy,” “epsilon wave,” “Fontaine lead,” “Lewis lead,” “torso ECG,” “non‐standard lead,” “alternative lead placement,” and “electrocardiography” or “ECG.” Reference lists of key articles and contemporary reviews were also screened to identify additional relevant reports.

We included peer‐reviewed human studies and case reports that described a clearly defined alternative ECG lead position or torso‐ECG configuration; and provided either quantitative diagnostic performance data (e.g., sensitivity, specificity, incremental diagnostic yield) or a clearly documented impact on clinical diagnosis compared with the conventional 12‐lead ECG. We excluded non‐peer‐reviewed material, conference abstracts without full data, animal or purely modeling studies, and reports lacking extractable diagnostic or clinical outcomes.

All titles and abstracts identified by the search were screened, and potentially eligible full‐text articles were reviewed in detail. From the final set of eligible publications, we extracted: underlying clinical setting (e.g., Brugada syndrome, ARVD/C, supraventricular tachycardia, acute coronary syndrome), description of the alternative configuration, number of patients included, number of ECG recordings analyzed (when reported), and main diagnostic performance metrics or incremental diagnostic yield compared with the standard 12‐lead ECG. Studies that provided quantitative comparative data are summarized in Table 1; additional supportive reports are cited in the text but not tabulated.

**TABLE 1:** Key studies evaluating alternative ECG lead placements.

First author, year	Clinical setting	Alternative configuration	Patients (*n*)	ECGs analyzed	Main comparative finding vs. standard 12‐lead ECG
Shimizu et al., 2000	Brugada syndrome (diagnostic ST pattern)	High right precordial leads (V1–V3 at 3rd–2nd intercostal spaces) + 87‐lead BSPM	25 Brugada + 40 controls	Not explicitly reported (multiple per patient)	High right precordial positions increased the proportion of Brugada patients with a diagnostic coved ST‐segment elevation pattern compared with standard V1–V3, without inducing type‐1 patterns in controls.
Hunuk et al., 2013	Brugada‐type ECG pattern in healthy men	Serial V1–V3 recordings at 4th, 3rd and 2nd intercostal spaces	504 healthy volunteers	1512 ECGs (3 per subject)	Brugada‐type ECG pattern increased from 3.0% (standard) to 5.0% (3rd ICS) and 7.5% (2nd ICS); type‐1 patterns were only seen at higher intercostal spaces, demonstrating higher sensitivity of high right precordial leads.
Gottschalk et al., 2014	ARVD/C—epsilon wave detection	Fontaine leads (bipolar precordial configuration over RVOT)	1 illustrative ARVD/C case	Standard + Fontaine ECGs in a single patient	Epsilon waves that were subtle or inapparent in standard precordial leads became clearly evident in Fontaine leads, illustrating increased sensitivity for ARVD/C‐related depolarization abnormalities.
Huemer et al., 2016	Wide‐QRS tachycardia—VA conduction type	Lewis (S5) lead added to standard 12‐lead ECG	Patients with wide‐QRS tachycardia (prospective series)	At least one tachycardia ECG per patient	Adding the Lewis lead significantly improved detection of ventriculoatrial conduction type and VA dissociation compared with the standard 12‐lead ECG alone, enhancing diagnostic accuracy for tachycardia mechanism.
Gurunath et al., 2021	Acute coronary syndrome—torso ECG	Torso‐ECG: limb electrodes relocated to torso; precordial leads unchanged	1361 chest‐pain patients (230 ACS; 324 with normal coronaries in main comparative cohort)	341 ACS ECG sets +324 normal‐coronary ECG sets (665 paired torso vs. standard ECGs)	Torso‐ECG showed very high concordance with conventional 12‐lead ECG for detection and localization of ischemic ST‐segment deviation in ACS, with no clinically relevant loss of diagnostic information.

## 3 Results

Several original clinical studies and illustrative reports were identified that evaluated alternative ECG lead positions or torso‐ECG systems in specific clinical settings. Among these, five key studies provided sufficiently detailed, extractable quantitative data on patient numbers and diagnostic performance of alternative lead configurations versus the conventional 12‐lead ECG. These studies, spanning Brugada‐pattern detection, ARVD/C, supraventricular tachycardia, and acute coronary syndromes, are summarized in Table 1.

### 3.1 Alternative ECG Position for the Detection of Brugada Syndrome

**FIGURE 1:**
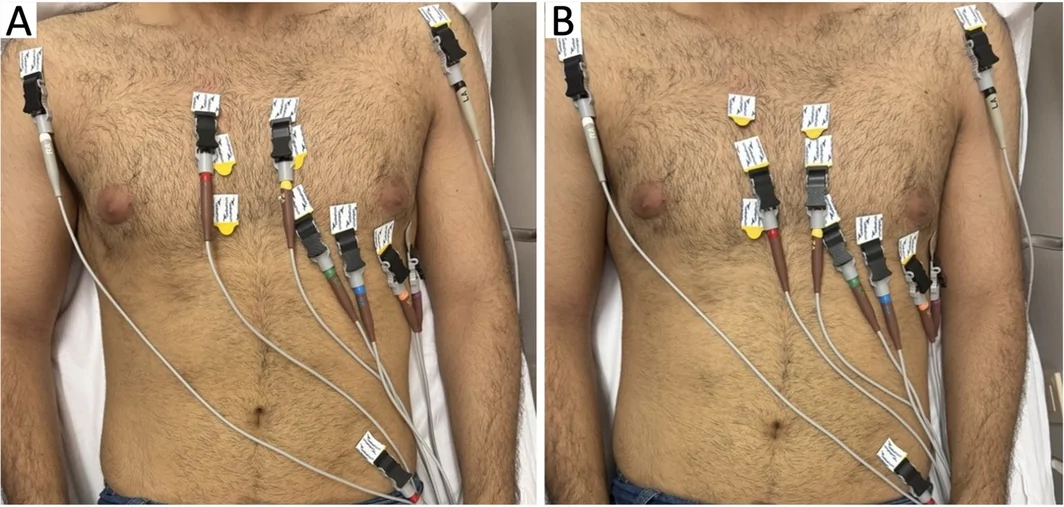
(A) Shimizu modification with V1 and V2 repositioned to the second intercostal space to enhance Brugada syndrome detection. (B) Standard 12‐lead ECG positioning for comparison.

The Shimizu lead placement represents a significant advancement in ECG, particularly in the detection of Brugada Syndrome, a condition often elusive to standard 12‐lead ECGs (Shimizu et al. 2000) (Figure 1). This innovative method involves repositioning the V1 and V2 electrodes to the 2nd intercostal space, a departure from their traditional 4th intercostal space location. This strategic placement, closer to the epicardial area of the right ventricular outflow tract, enhances the detection of the syndrome's characteristic ST elevations, thereby improving the identification of this often‐missed condition (Shimizu et al. 2000). The accuracy of electrode placement is vital, as it directly impacts the ability to detect the critical electrophysiological markers of Brugada Syndrome (Shimizu et al. 2000; Serra et al. 2014) (Figure 1).

The clinical impact of the Shimizu lead placement could be profound. It ensures a more sensitive detection of Brugada Syndrome, facilitating earlier diagnosis and more precise risk stratification, which is crucial for improving patient outcomes (Shimizu et al. 2000; Serra et al. 2014). This approach is particularly relevant in cases where standard ECG readings are ambiguous and there is still a suspicion of Brugada Syndrome, indicated by symptoms such as unexplained syncope or a family history of sudden cardiac death (Baranchuk et al. 2015).

### 3.2 Alternative ECG Lead Placement for Diagnosis of Arrhythmogenic Right Ventricular Dysplasia (ARVD)

**FIGURE 2:**
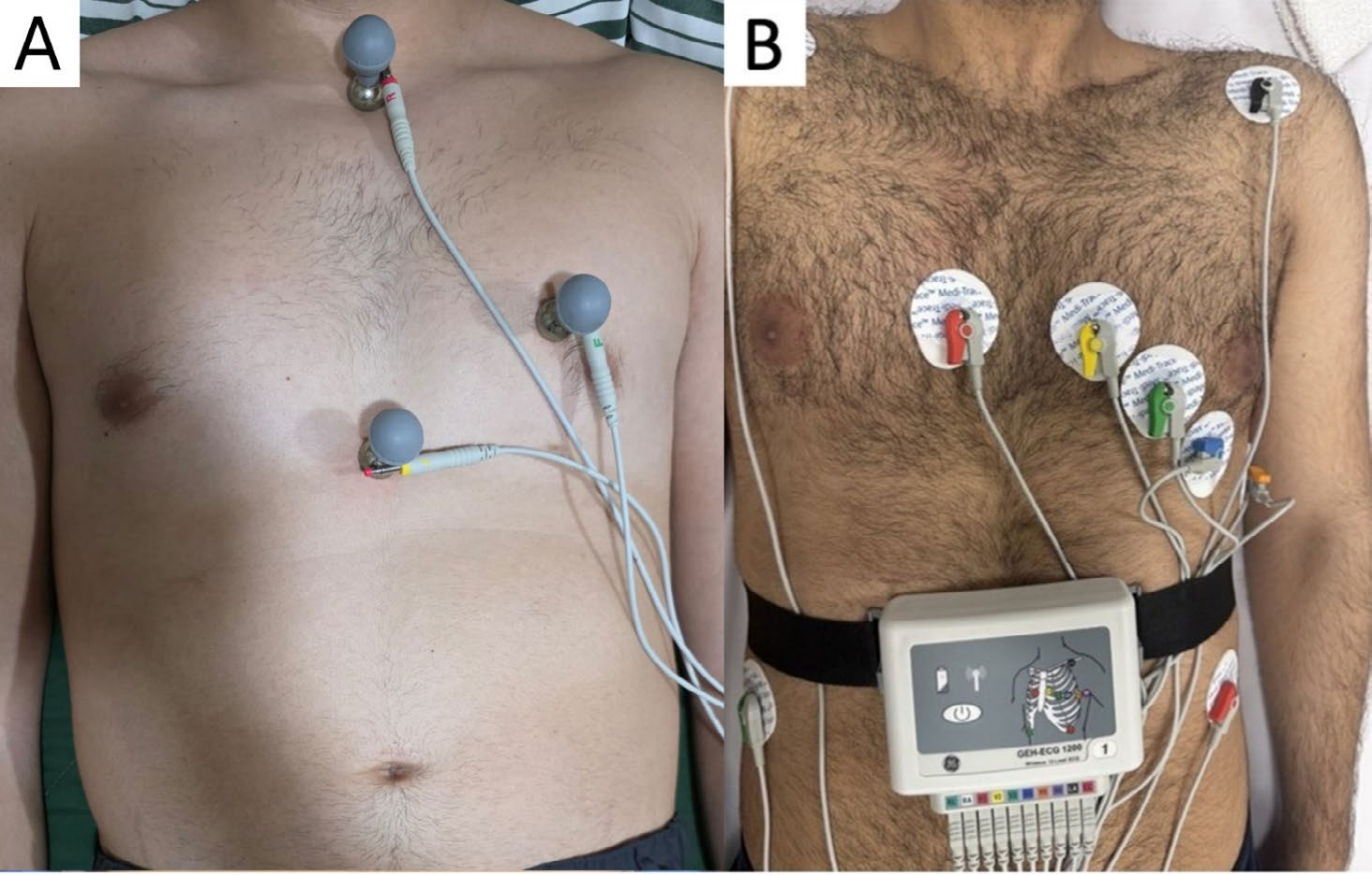
(A) Fontaine lead placement to improve detection of arrhythmogenic right ventricular dysplasia/cardiomyopathy (ARVD/ARVC). (B) Modified exercise‐test configuration to reduce motion artifact and maintain ECG fidelity during exertion.

Fontaine lead placement is primarily used in the evaluation of Arrhythmogenic Right Ventricular Dysplasia/Cardiomyopathy (ARVD/C), a progressive disorder characterized by fibro‐fatty replacement of the right ventricular myocardium (Figure 2A). This modified configuration enhances detection of ECG features, including epsilon waves, localized QRS prolongation, and T‐wave inversion, that may be subtle or absent on standard 12‐lead recordings, thereby improving diagnostic sensitivity (Gottschalk et al. 2014).

The Fontaine lead placement is a modified ECG technique designed to improve the recording of electrical activity from the right ventricle, which is often underrepresented in standard 12‐lead ECGs due to its reduced muscle mass and the limb leads' predominant orientation toward the left ventricle (Gottschalk et al. 2014; Peters and Trümmel 2003). In this method, the conventional limb electrodes are repositioned: the right arm electrode is placed on the manubrium, the left arm electrode over the xiphoid process, and the left leg electrode at the standard V4 position (Figure 2A) (Gottschalk et al. 2014; Peters and Trümmel 2003). This configuration produces three bipolar chest leads (F I, F II, F III) that accentuate right ventricular electrical potentials. By enhancing signal amplitude from the right ventricle, Fontaine lead placement can improve the detection of characteristic abnormalities, including epsilon waves, localized QRS prolongation, and T‐wave inversion, which are key ECG markers of ARVD/C. This approach is most often used when ARVD/C is suspected and the standard ECG is nondiagnostic, and it has been shown in several studies to increase diagnostic yield for right ventricular electrical abnormalities (Gottschalk et al. 2014; Peters and Trümmel 2003).

### 3.3 Alternative Lead Placement for Stress Testing in Cardiac Diagnostics

In ECG stress testing, lead placement is a critical factor in ensuring accurate detection of ischemic heart disease. The exercise ECG aims to monitor the heart's electrical activity under physical exertion, revealing abnormalities that may not be evident at rest (Fletcher et al. 2013). While the lead configuration typically mirrors that of the standard 12‐lead ECG, minor adjustments are often necessary to optimize the stability of electrode connections and maintain high‐quality signal recordings during exercise (Figure 2B) (Fletcher et al. 2013).

In most laboratories, a Mason‐Likar‐type torso configuration is used. In this approach, the chest leads (V1–V6) remain in their standard positions, with V1 and V2 placed on either side of the sternum in the 4th intercostal space, and V3–V6 extending from the mid‐clavicular line toward the anterior and mid‐axillary lines. The limb electrodes, which are typically placed on the wrists and ankles in a resting ECG, are shifted to the torso to reduce motion artifact from moving extremities (Fletcher et al. 2013). The right and left arm electrodes (RA and LA) are positioned just below the clavicles, while the right and left leg electrodes (RL and LL) are placed on the lower torso near the hips (Figure 2B) (Fletcher et al. 2013).

Relocating the limb electrodes to the torso can produce small, configuration‐dependent shifts in QRS axis and baseline ST‐segment level compared with a fully standard, limb‐based 12‐lead ECG, particularly when posture also changes between rest and exercise (Madias 2006). However, when the Mason‐Likar configuration is applied consistently throughout the stress protocol and a short resting tracing is obtained using the same setup before exercise, these differences do not typically preclude the detection of clinically relevant horizontal or down sloping ST‐segment depression (Fletcher et al. 2013; Madias 2006). In this context, the modified stress‐test lead arrangement should be regarded as a pragmatic technical adaptation of the standard 12‐lead ECG that prioritizes signal stability without abandoning the fundamental principles of electrocardiographic interpretation (Fletcher et al. 2013; Madias 2006).

In addition to the Mason‐Likar modification, the Lund lead system is a torso‐based limb‐lead configuration intended to reduce motion artifact by relocating limb electrodes to more proximal sites. In practice, it aims to preserve a stable limb‐lead axis while improving signal quality during monitoring or exercise and when standard distal limb placement is impractical (e.g., limb amputations, extensive dressings, burns, tremor, or severe movement). As with any torso‐based limb‐lead relocation, small shifts in frontal‐plane QRS axis and baseline ST‐segment level may occur compared with a conventional resting 12‐lead ECG; therefore, the same configuration should be used consistently for serial recordings, and the tracing should clearly document the lead system used (Pahlm and Wagner 2008a).

### 3.4 Seated ECG Protocol for Cardiovascular Assessment in Patients Who Can't Tolerate Supine Position

**FIGURE 3:**
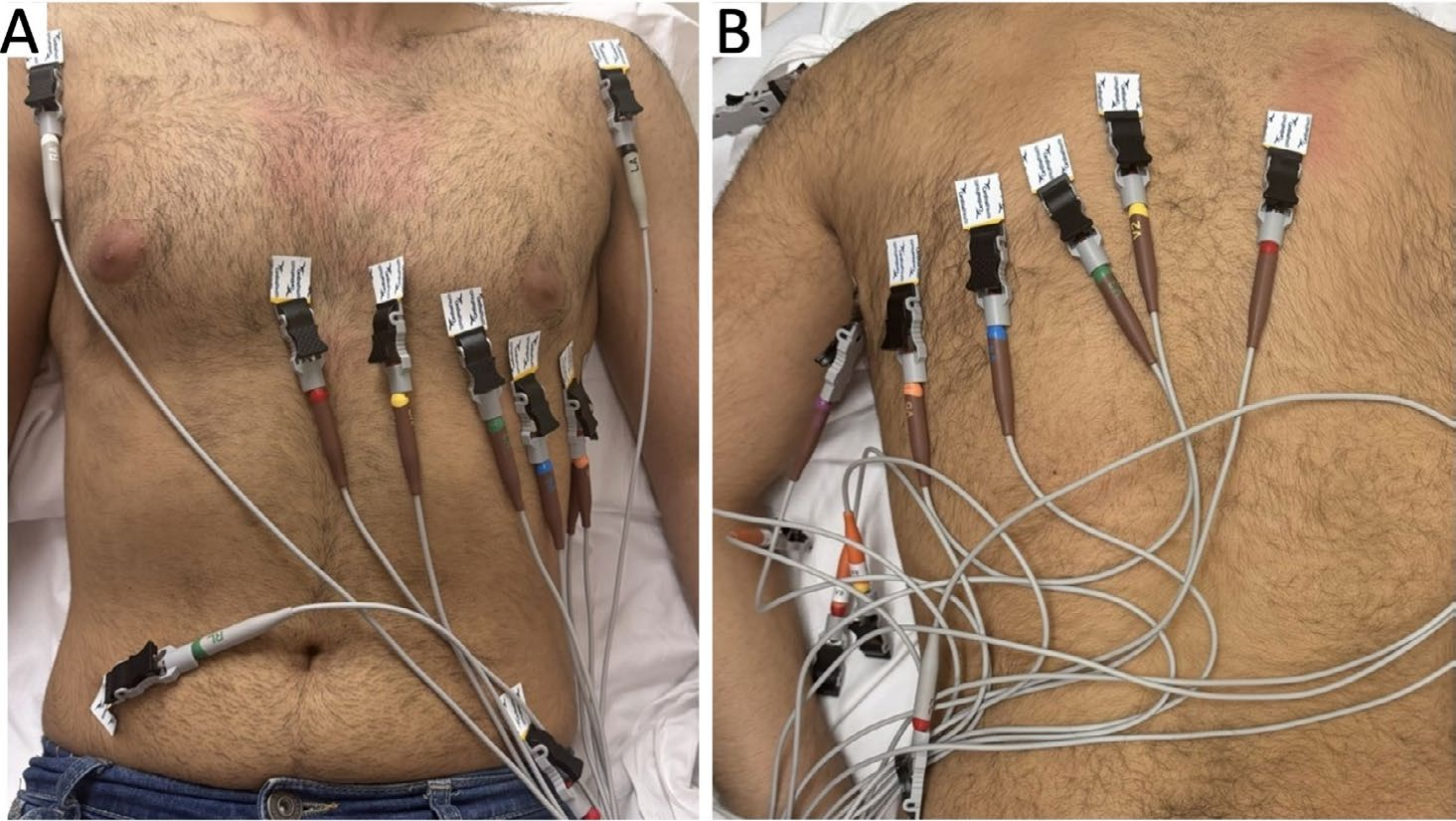
(A) Seated 12‐lead ECG for patients unable to tolerate the supine position. (B) Prone lead placement for ECG acquisition in prone‐positioned patients.

The seated 12‐lead ECG is a practical adaptation of the standard ECG protocol for patients who cannot tolerate the supine position because of pain, dyspnea, recent surgery, bulky dressings, spine disease, frailty or other physical limitations (Figure 3A). This situation is common in a wide range of clinical settings, including oncology (e.g., after thoracic or abdominal procedures), advanced heart failure, severe orthopnea and musculoskeletal disorders. In such patients, insisting on a fully supine position may be uncomfortable, unsafe or simply not feasible; a seated ECG offers a patient‐centered alternative that still provides diagnostically useful information (Baranchuk et al. 2009).

The basic principle of the seated ECG is to maintain the standard anatomical positions of the precordial leads while adapting the patient's posture and, when necessary, the placement of limb electrodes. Thus, V1–V6 are placed as in a conventional 12‐lead ECG on the anterior chest wall: V1 and V2 parasternal in the 4th intercostal space, V4 in the 5th intercostal space at the mid‐clavicular line, and V3, V5, and V6 interpolated along the anterior and mid‐axillary lines (Figure 3A). Limb electrodes (RA, LA, RL, LL) can be attached to the wrists and ankles as usual, or if access is limited, to more proximal sites on the upper arms and thighs or to the lower torso, provided that they are placed symmetrically and sufficiently distant from the precordial leads to avoid overlap of recording fields (Baranchuk et al. 2009; Madias 2006). The patient should be seated upright with adequate back support and feet on the floor to minimize motion artifact and maintain a stable recording position.

From a physiological standpoint, changes in posture from supine to seated may modestly increase heart rate and alter autonomic tone, which can lead to small variations in PR, QRS, and QT intervals and in T‐wave morphology (Madias 2006). These differences must be considered when interpreting the tracing and when comparing it with prior or subsequent supine ECGs. The ECG should clearly state in the header that it was acquired in the seated position. Despite these nuances, seated 12‐lead ECGs generally provide reliable information for the detection of rhythm disturbances, conduction abnormalities, marked axis deviation, and overt ischemic changes (Baranchuk et al. 2009; Madias 2006). In patients who cannot safely lie supine, this configuration allows timely cardiac assessment and surveillance for treatment‐related cardiotoxicity or decompensation without adding to the burden imposed by their underlying condition.

### 3.5 Prone ECG

The prone 12‐lead ECG is a practical adaptation for patients who must remain in the prone position, particularly those with acute respiratory distress syndrome (ARDS) receiving prone ventilation to improve oxygenation (Figure 3B). In this setting, obtaining a standard supine 12‐lead ECG is often impractical or unsafe because turning the patient may compromise respiratory support, vascular access, or hemodynamic stability. A prone ECG configuration allows continuous rhythm and ST‐T monitoring without repeated repositioning and can therefore support ongoing cardiac assessment in critically ill patients (Roccia et al. 2021).

In a prone ECG setup, precordial leads are repositioned onto the posterior thorax while limb leads remain attached in their usual locations or to proximal limb/torso sites to minimize dislodgement. In the configuration most frequently described, V1 and V2 are placed paraspinally on either side of the spine, and V3–V6 are moved laterally across the back to sample the lateral and inferior myocardial territories, while preserving a roughly analogous spatial distribution to the anterior chest arrangement (Figure 3B). This adaptation enables monitoring of global ST‐segment trends, T‐wave morphology, and gross myocardial injury patterns in patients ventilated in the prone position (Roccia et al. 2021).

Clinical series in ARDS due to COVID‐19 have shown that prone ECG tracings are generally adequate for the detection of significant rhythm disturbances, conduction abnormalities, and evolving inferior or lateral ST‐segment changes (Haseeb et al. 2020). However, because of the altered lead vectors, sensitivity for purely anterior ischemia is reduced, and ST‐segment amplitudes may not be directly comparable with standard supine recordings (Haseeb et al. 2020). For this reason, when hemodynamically feasible, suspicious abnormalities identified on prone ECG should be confirmed with a conventional supine 12‐lead ECG. In routine practice, prone ECG should therefore be viewed primarily as a complementary monitoring tool for high‐risk, ventilated patients, rather than a full substitute for standard ischemia‐focused ECG assessment (Roccia et al. 2021; Haseeb et al. 2020).

### 3.6 Posterior ECG Leads for Enhanced Detection of Lateral Myocardial Infarction

**FIGURE 4:**
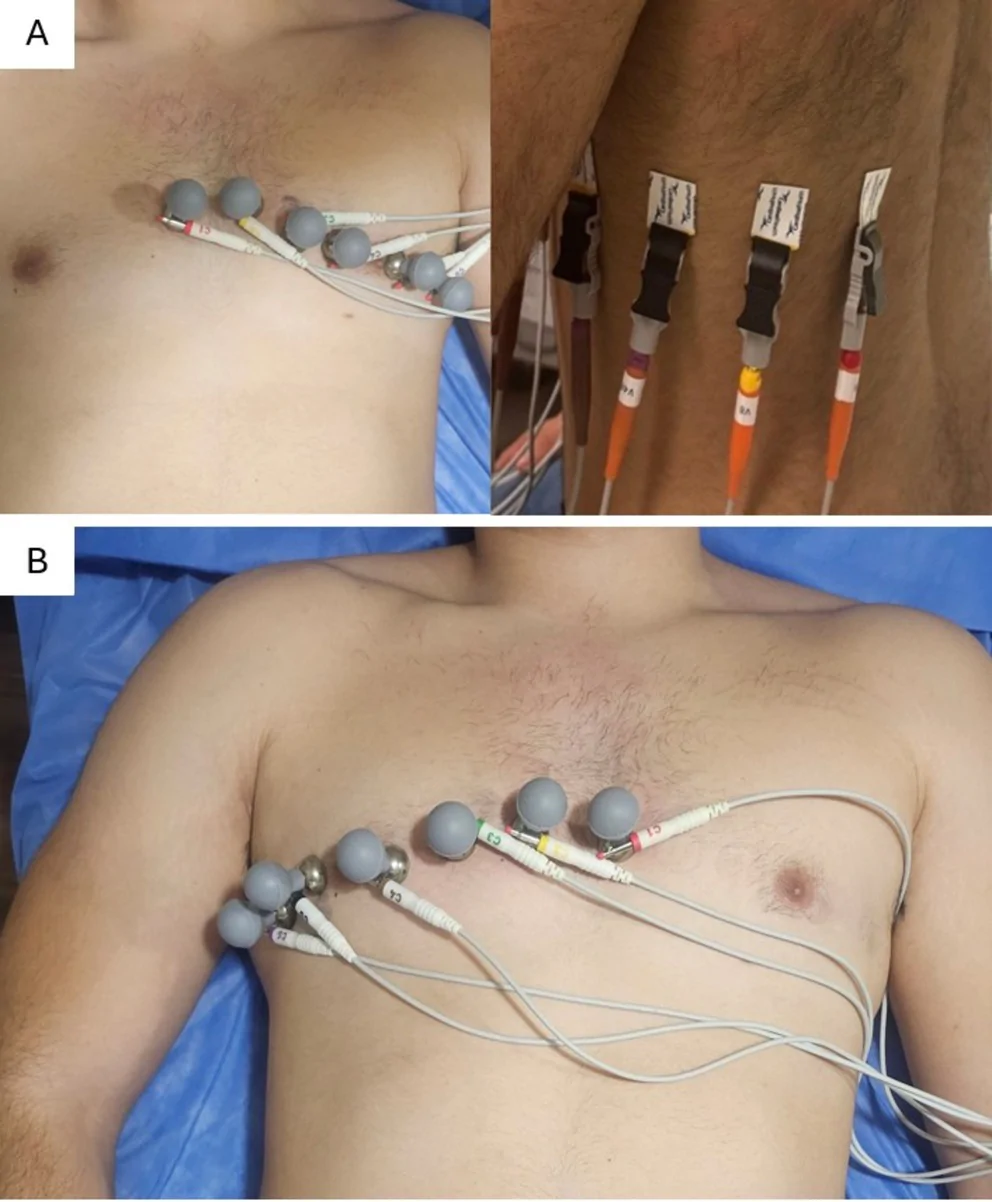
(A) Posterior chest leads V7–V9 to improve detection of lateral/inferolateral ischemia. (B) Right‐sided precordial leads V1R–V6R for identification of right ventricular myocardial infarction (RVMI).

The use of posterior ECG leads is essential in detecting lateral myocardial infarction, particularly when standard 12‐lead ECGs fail to reveal ischemic changes in the lateral or inferolateral walls of the heart. Standard ECGs primarily view the anterior and inferior regions of the heart, often missing critical ischemic changes in the inferolateral or lateral regions. By adding posterior leads (V7, V8, V9), clinicians can gain a more complete view of the heart's electrical activity, improving the accuracy of lateral myocardial infarction diagnosis (Figure 4A) (Smith 2006).

As shown in Figure 4A, the posterior leads are positioned as follows: V1–V6 are positioned similarly to the standard 12 lead ECG, V7 is located at the left posterior axillary line, V8 is positioned at the left mid‐scapular line, and V9 is placed at the left paraspinal line, all at the same horizontal level as V4 (Smith 2006; Bischof et al. 2018). These additional leads provide a more sensitive detection of ischemic changes in the inferolateral and lateral myocardium. This is particularly important when diagnosing lateral myocardial infarction, which often involves the circumflex artery and may not show clear signs of ischemia in the standard precordial leads (Smith 2006). Early detection of lateral MI using posterior leads allows for timely interventions, specially in cases where patients exhibit atypical symptoms or the standard ECG results are inconclusive.

A practical 15‐lead ECG approach may also be used by adding V4R together with posterior leads V8 and V9 to the standard 12‐lead ECG, particularly in inferior STEMI when right ventricular involvement is suspected and when inferobasal extension is a concern, as this may increase diagnostic yield with minimal additional acquisition time (Rao et al. 2025).

### 3.7 Alternative ECG Position for the Detection of Right Ventricular Abnormalities (Right Precordial Leads)

Right Precordial leads are vital for detecting conditions such as right ventricular myocardial infarction (RVMI) and Brugada Syndrome, conditions that are often missed by 12‐lead ECG (Figure 4B) (Koppikar et al. 2015). Incorporating right‐sided leads provides a more comprehensive cardiac assessment, capturing electrical activity from the right ventricle and leading to a more accurate diagnosis (Hunuk et al. 2013).

Right Precordial leads are positioned in a mirror image of the standard left‐sided leads. As illustrated in Figure 4B, in a mirror‐image right‐sided precordial acquisition: lead V1R is placed in the 4th intercostal space at the left sternal border, mirroring the standard placement of V1; lead V2R is placed in the 4th intercostal space at the right sternal border (Hunuk et al. 2013; Pahlm and Wagner 2008b). Subsequent leads, from V3R to V6R, follow along the right side of the chest, with V6R ending at the right midaxillary line. This placement provides enhanced sensitivity to right ventricular electrical activity, which is often underrepresented by standard ECG leads (Hunuk et al. 2013; Pahlm and Wagner 2008b).

Right precordial leads are particularly useful in diagnosing RVMI, where they can reveal ST‐segment elevation that might not appear in the standard leads. Similarly, in Brugada Syndrome, the characteristic ECG pattern (right bundle branch block and ST‐segment elevation in the right precordial leads) is best detected by these leads. Studies have shown that including these right‐sided leads significantly improves the diagnostic yield in these conditions leading to timely interventions (Baranchuk et al. 2009; Pahlm and Wagner 2008b).

Right‐sided precordial lead acquisition is also routinely used in patients with suspected dextrocardia, typically by recording the precordial leads in a mirrored configuration over the right hemithorax (often labeled V1R–V6R) and clearly documenting this acquisition on the tracing to avoid misinterpretation (Yoo et al. 2021).

### 3.8 Modified Lewis Lead

**FIGURE 5:**
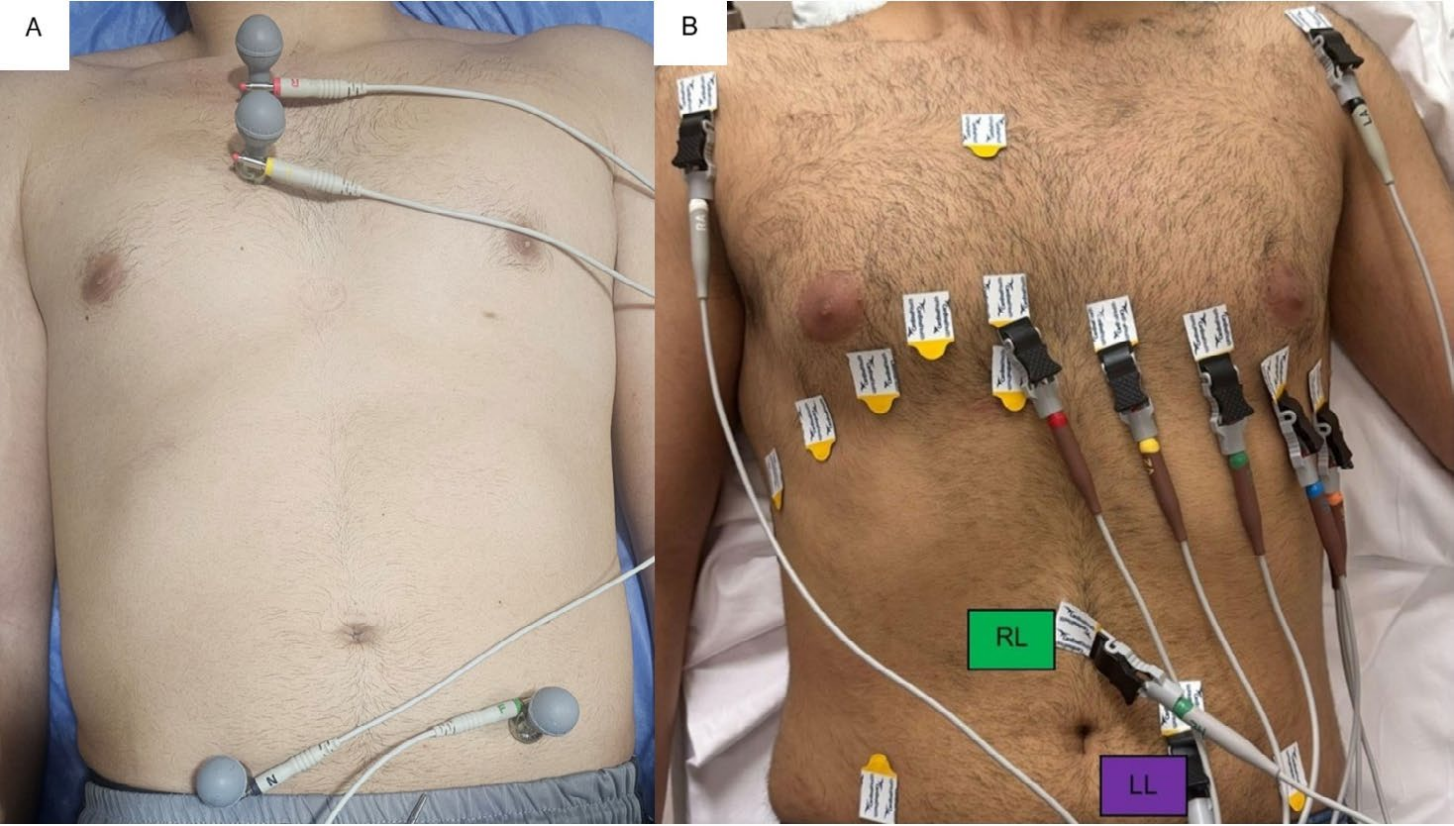
(A) Modified Lewis lead to enhance atrial arrhythmia visualization. (B) Torso ECG (T‐ECG) with centralized electrodes for rapid application in emergency or prehospital settings.

The Modified Lewis Lead, or S5 lead, is an alternative ECG configuration primarily utilized for the detection of atrial arrhythmias, such as atrial flutter and atrial tachycardia (Pachón et al. 2019). Further, this lead is helpful in identifying atrioventricular dissociation in the setting of wide QRS complex tachycardias, one crucial diagnostic finding that differentiates ventricular from supraventricular tachycardias (Figure 5A). Contrary to the regular ECG, where ventricular activity may mask the signals of the atria, the special positioning in the Lewis Lead aims at amplification of the atrial activity and at diminishing the impact of the QRS complex (Pachón et al. 2019; Surawicz and Knilans 2009). Though application to large clinical trials has still not widely been executed, small sample size studies have shown its effectiveness in cases of emergencies and certain arrhythmia management scenarios. For instance, Huemer et al. established that the Modified Lewis Lead configuration significantly improved the detection of atrial signals, particularly during ventricular pacing (Pachón et al. 2019; Surawicz and Knilans 2009).

To set up the Modified Lewis Lead, as shown in Figure 5A, the RA electrode is repositioned to the second intercostal space, parasternal right, while the LA electrode is placed in the fourth intercostal space, also on the right side of the sternum (Pachón et al. 2019; Surawicz and Knilans 2009; Huemer et al. 2016). The LL electrode remains in its standard position on the lower left torso. This configuration increases the gain of atrial depolarization signals by aligning the electrical axis along the path of atrial depolarization, thus suppressing ventricular interference (Pachón et al. 2019; Surawicz and Knilans 2009; Huemer et al. 2016). The modified electrode placement in the figure shows better visualization of P‐waves and thus better identification of conduction at the time of arrhythmias. Indeed, the Lewis Lead configuration in the study conducted by Huemer et al. achieved a strikingly higher percentage of correctly detected atrioventricular dissociation (75% in the Modified Lewis Lead group compared to 62% in the group analyzed by a standard‐lead ECG), emphasizing its diagnostics further (Antiperovitch et al. 2019). The enhanced capacity of the Modified Lewis Lead configuration to isolate atrial electrical activity renders it particularly valuable in clinical scenarios where standard ECG lead placements fail to provide adequate diagnostics for atrial arrhythmias and fail to differentiate atrial and ventricular arrhythmias, especially in acute or time‐sensitive settings (Pachón et al. 2019; Surawicz and Knilans 2009; Huemer et al. 2016).

### 3.9 Alternative ECG Position for the Detection of Cardiac Abnormalities in Torso‐Based Recordings

A torso ECG (T‐ECG) uses electrodes placed exclusively on the torso, spanning the anterior, posterior, and lateral sides of the body between the neck and the pelvis (Figure 5B). Its efficacy in diagnosing acute coronary syndrome (ACS) has been evaluated in comparison with the conventional ECG (C‐ECG) electrode positions, which are considered the benchmark for 12‐lead interpretation (Gurunath et al. 2021; Khan 2015).

The T‐ECG model proposes a more convenient positioning of the limb leads, concentrating them in a central thoracoabdominal area while leaving the precordial leads unchanged. This configuration was originally conceived for pre‐hospital and in‐hospital settings (ambulances, emergency departments, chest‐pain units), where rapid application, easier access to the extremities and reduced cable clutter are important (Madias 2006; Gurunath et al. 2021). By keeping all electrodes on the torso, T‐ECG can be applied quickly even in immobile, obese, or critically ill patients and facilitates serial recordings during the evolution of chest pain. In the longer term, similar torso‐based layouts may inform the design of wearable or semi‐permanent multilead devices for remote monitoring, although fully self‐applied 12‐lead ECG systems for routine home use are not part of current standard clinical practice (Pahlm and Wagner 2008b; Gurunath et al. 2021; Khan 2015).

Optimizing time is crucial in ACS because it can rapidly progress to life‐threatening complications such as malignant arrhythmias and heart failure (Rao et al. 2025). In a comparative study, after careful testing of the T‐ECG against the conventional 12‐lead ECG, this alternative model performed as accurately as the C‐ECG for the diagnosis and classification of ACS (Gurunath et al. 2021). The T‐ECG successfully identified all 341 ECG sets diagnosed with ACS by the C‐ECG, as well as all 324 ECGs that were classified as normal by the C‐ECG. Furthermore, the 234 ACS ECGs with ST elevation and the 154 ACS ECGs containing ST depression were all correctly detected by the T‐ECG (Gurunath et al. 2021). These data suggest that, when properly applied, T‐ECG can reproduce the diagnostic information of the standard 12‐lead ECG in the acute setting (Gurunath et al. 2021).

The configuration of the 12‐lead T‐ECG is shown in Figure 5B. The traditional anterior chest ECG leads (V1–V6) remain unchanged in this model, as do the V7–V9 leads conventionally placed on the left posterior chest wall (Smith 2006; Bischof et al. 2018). Electrodes placed on the upper extremities are repositioned to the deltopectoral groove on the corresponding side of the body, along the lateral border of the clavicle. The LL electrode is positioned two finger‐widths to the left of the umbilicus, and the RL electrode two finger‐widths directly above the umbilicus (Gurunath et al. 2021; Khan 2015). This strategic alternative offers a quicker and more intuitive placement of the leads, which can improve workflow efficiency and facilitate timely ECG acquisition in patients presenting with chest pain (Gurunath et al. 2021; Khan 2015).

## 4 Discussion

This study highlights the importance of alternative ECG lead placements for strengthening the diagnostic accuracy of cardiac diseases. They offer more flexibility in assessing conditions by expanding the location of the leads and improving sensitivity, especially for complex diseases in which ECG diagnosis is challenging. Although the traditional 12‐lead ECG remains widely used in clinical practices, limitations exist when assessing abnormalities in the right ventricle and inferolateral wall which can be resolved by the use of alternative leads (Baranchuk et al. 2009).

For example, the Shimizu lead placement is helpful in the detection of Brugada Syndrome, often missed by the 12‐lead ECG (Shimizu et al. 2000). The repositioning of the V1 and V2 to the intercostal space allows for specific detection of the ST‐segment elevations that would otherwise go unnoticed (Shimizu et al. 2000). The Fontaine lead placement is specific to the diagnosis of ARVD through detection of epsilon waves and conduction delays (Gottschalk et al. 2014). Posterior (V7–V9) and right precordial (V1R–V6R) alternative leads are crucial for the detection of myocardial infarctions, including inferolateral, lateral, and right ventricular because the relocations of these leads are tailored for sensing specific electrical activity required to identify these conditions (Smith 2006; Bischof et al. 2018).

Furthermore, the prone ECG is optimized to treat patients with ARDS or COVID‐19 using the prone position and avoids the need for repositioning (Roccia et al. 2021; Haseeb et al. 2020). The Lewis lead is adapted for diagnosing atrial arrhythmias through specific detection of P‐wave abnormalities when atrial fibrillation is present (Pahlm and Wagner 2008b; Yoo et al. 2021; Pachón et al. 2019; Surawicz and Knilans 2009).

Alternative lead placements also offer stress testing functionalities with modifications to isolate for ischemia and coronary artery disease. Seated ECG readings are another application of alternative ECG leads that allow patients unable to lie down to receive more accurate diagnoses, adapted to their needs (Madias 2006).

These expanded lead positions, while enhancing diagnostic capability, also introduce a higher potential for misplacement and interpretation error (Baranchuk et al. 2009). On the positive side, they can substantially increase diagnostic sensitivity in selected conditions thus offering practical tools that complement conventional ECG. At the same time, their use is not without drawbacks: non‐standardized placement protocols, operator variability, and lack of widespread familiarity may reduce reproducibility and create opportunities for both over‐ and under‐diagnosis. From a clinical standpoint, these techniques should therefore be applied selectively, in situations where they clearly add value to patient assessment and always interpreted within the broader clinical and electrophysiological context. Moving forward, proper training and standardization will be critical to minimize inaccuracies, while further research is needed to validate these methods in larger populations, refine their technical implementation, and explore integration with emerging digital tools.

## 5 Limitations

While this narrative review draws upon a wide array of published reports to illustrate the clinical utility of alternative ECG lead placements, it is subject to several limitations. First, our selection of studies was guided by clinical relevance and consequently, relevant reports (particularly those with negative or inconclusive findings) may have been overlooked. Second, the methodologies and patient cohorts described in the literature vary considerably in design, setting, and sample size, ranging from small, single‐center series to larger cohorts, thus hindering direct comparison of diagnostic performance across different lead configurations. Third, there is no standardization in how these alternative lead positions are implemented: intercostal spacing, patient posture, electrode fixation, and even ECG machine calibration differs from one report to the next, and few authors have evaluated inter‐operator reproducibility or the learning curve required to achieve reliable placement.

Moreover, the existing literature tends to emphasize technical parameters such as sensitivity, specificity, and waveform clarity over clinically meaningful endpoints such as changes in patient management, morbidity, or mortality. Practical considerations, including the additional time required to apply non‐standard leads, the need for extra consumables, and potential software or hardware modifications, have not been systematically assessed in real‐world workflows. Finally, by relying predominantly on peer‐reviewed publications that highlight diagnostic “successes,” there is a risk of publication bias inflating the perceived benefit of certain lead modifications.

## 6 Conclusion

The ECG remains an essential tool in clinical practice, and understanding alternative lead placements can enhance its diagnostic value in specific clinical scenarios. By recognizing the limitations of standard 12‐lead ECGs and the benefits of tailored lead adjustments, clinicians can improve diagnostic accuracy, avoid misinterpretations, and make more informed therapeutic decisions. Familiarity with these alternative placements allows healthcare providers to adapt ECG recordings for complex cases, ensuring a more comprehensive assessment and better patient outcomes.

## Author Contributions

Conceptualization: José Luis Morales‐Arteaga, Amin Meghdadi, Adrian Baranchuk. Literature review and manuscript drafting: José Luis Morales‐Arteaga, Amin Meghdadi, Derek Wu, Abdullah Bhuiyan, Nicole Langleben, Cengiz Burak, Emily Wilson, Ashley Pellerito, David Aristizábal, Keanu Razzaghi, Juan Maria Farina, Shyla Gupta. Critical revision of the manuscript: José Luis Morales‐Arteaga, Amin Meghdadi, Juan Maria Farina, Shyla Gupta, Adrian Baranchuk. Supervision: José Luis Morales‐Arteaga, Amin Meghdadi, Adrian Baranchuk. All authors reviewed and approved the final version of the manuscript.

## Funding

The authors have nothing to report.

## Conflicts of Interest

The authors declare no conflicts of interest.

## Data Availability

The data that support the findings of this study are available from the corresponding author upon reasonable request.
